# Estudo Comparativo entre Receptores de Desfibriladores Subcutâneos e Transvenosos em Relação à Tolerância ao Procedimento de Implante e Percepção da Qualidade de Vida

**DOI:** 10.36660/abc.20190312

**Published:** 2021-06-08

**Authors:** Pablo Esteban Auquilla-Clavijo, Naiara Calvo-Galiano, Marina Povar-Echeverría, Teresa Oloriz-Sanjuan, Francisco Diaz-Cortejana, Antonio Asso-Abadia

**Affiliations:** 1 Hospital Universitario Miguel Servet Zaragoza Espanha Hospital Universitario Miguel Servet – Cardiologia, Zaragoza - Espanha; 2 Hospital Universitario Miguel Servet Zaragoza Espanha Hospital Universitario Miguel Servet -Medicina Interna, Zaragoza - Espanha

**Keywords:** Desfibriladores Implantáveis, Desfibriladores Implantáveis Subcutâneos, Estudo Comparativo, Sedação Consciente, Qualidade de Vida

## Abstract

**Fundamento:**

O cardioversor-desfibrilador implantável subcutâneo (S-CDI) é uma alternativa segura ao sistema convencional do CDI transvenoso (TV-CDI) para evitar morte súbita.

**Objetivo:**

Comparar o impacto do tipo de sistema de CDI e técnica cirúrgica na qualidade de vida dos pacientes, bem como a gravidade do desconforto e da dor, entre receptores de S-CDI e TV-CDI.

**Métodos:**

Pacientes consecutivamente implantados com um sistema S-CDI foram pareados com pacientes com um sistema TV-CDI. Além disso, foram incluídos os pacientes submetidos ao implante de S-CDI após a remoção de um TV-CDI, devido a complicações. Foram avaliadas a qualidade de vida (medida com o questionário de saúde de 12 itens) e a gravidade da dor e desconforto. Significância estatística foi definida como p < 0,05.

**Resultados:**

Foram analisados 64 pacientes implantados com S-CDI ou TV-CDI sob anestesia local e sedação consciente. Pacientes com sistemas S-CDI e TV-CDI não diferiram significativamente nos escores de qualidade de vida. Os pacientes com S-CDI apresentaram maior nível de dor peri-operatória; nenhuma diferença foi encontrada em relação à gravidade da dor intra-operatória. A magnitude do desconforto estético e dos distúrbios do sono não diferiu entre os grupos.O S-CDI foi implantado em 7 pacientes adicionais após a remoção de um TV-CDI. Todos exceto um desses pacientes recomendaram o sistema S-CDI.

**Conclusões:**

O tipo de sistema de CDI e a técnica cirúrgica têm impacto insignificante na qualidade de vida dos pacientes. Estes resultados sugerem que a sedação consciente, fornecida por uma equipe experiente de eletrofisiologia, pode ser considerada uma alternativa à anestesia geral para o manejo de pacientes submetidos ao implante de S-CDI.

## Introdução

A morte súbita cardíaca (MSC) de origem arrítmica é a principal causa de mortalidade cardiovascular. A eficácia do cardioversor-desfibrilador implantável (CDI) para reduzir a mortalidade por MSC em populações selecionadas tem sido amplamente demonstrada em diversos ensaios clínicos. ^1^ Os sistemas convencionais de desfibrilador consistem em um gerador de pulso localizado na área peitoral, conectado ao endocárdio por meio de eletrodos transvenosos. Este tipo de dispositivo é, portanto, sujeito a complicações inerentes ao mecanismo de implantação e à posição intravascular dos eletrodos.

Devido aos problemas de acesso ao coração pelo sistema venoso e ao potencial para complicações, foi desenvolvido o cardioversor-desfibrilador implantável subcutâneo (S-CDI, Boston Scientific, Natick, MA, EUA). Este sistema consiste em um gerador (S-ICD ^®^ , EMBLEM MRI S-ICD A219, Boston Scientific) conectado a um eletrodo (3401, Boston Scientific) localizado subcutaneamente em posição paraesternal, geralmente à esquerda. ^2^ As diretrizes clínicas atuais incluem o S-CDI, com indicação de Classe IIa, como alternativa ao CDI transvenoso (TV-CDI) convencional em pacientes que não requerem antibradicardia, antitaquicardia ou terapia de ressincronização. Ele também possui indicação de Classe IIb em pacientes sem acesso venoso, após a remoção de um sistema transvenoso devido a infecção, e em pacientes jovens que têm necessidade vitalícia de terapia baseada em dispositivo. ^2^ Tem sido relatada uma taxa limitada de complicações relacionadas ao implante. Além disso, embora não existam estudos comparativos randomizados de S-CDI versus TV-CDI até o momento, os dados disponíveis mostram que o S-CDI é um dispositivo muito eficaz para a detecção e o tratamento de arritmias ventriculares malignas. ^3 - 6^

O uso de S-CDI na Espanha é cada vez mais aceito pelas sociedades científicas. No estudo de Arias et al., ^4^ de 2017, em um centro espanhol, foi possível obter excelentes resultados agudos e de longo prazo em uma coorte de 50 pacientes com S-CDI. ^7^ O mais recente registro espanhol de implante de CDI, em 2017, indica um aumento progressivo nos implantes de S-CDI de 2,5% em 2015 para 5,3% em 2017. ^8^ O custo mais elevado do S-CDI em relação ao TV-CDI pode ser um dos motivos que a adoção desse dispositivo tem sido lenta, apesar de seu design revolucionário. ^9^ O desenvolvimento de estudos multicêntricos para apoiar estes resultados ^7^ nos permitiria ampliar a utilização do dispositivo.

Em termos da influência do implante de CDI na qualidade de vida, a literatura apresenta evidências contraditórias. ^10 - 17^ Enquanto as experiências iniciais de CDI foram associadas a pior qualidade de vida, estudos mais recentes demonstraram qualidade de vida, pelo menos, comparável a de pacientes da população geral, sem CDI. ^16 , 18^ Foi publicado recentemente o único estudo que avaliou e comparou a qualidade de vida em pacientes com TV-CDI versus S-CDI. Houve uma melhora na qualidade de vida em ambos os grupos de pacientes, conforme medido pela pesquisa de saúde SF-12, e nenhuma diferença significativa foi observada entre os grupos. ^19^

## Objetivo

O objetivo do nosso estudo foi o de comparar a qualidade de vida percebida, bem como a intensidade da dor e do desconforto, decorrente da técnica cirúrgica e do tipo de dispositivo, entre uma população de pacientes que receberam S-CDI e um grupo controle que recebeu TV-CDI convencional.

## Métodos

Foram consecutivamente inscritos todos os pacientes implantados com S-CDI em nosso hospital de 2014 a 2016. Os pacientes foram pareados por idade, sexo e índice de massa corporal com uma amostra de pacientes submetidos ao primeiro implante de TV-CDI de câmara única, sem indicação de terapia antibradicardia ou estimulação de antitaquicardia, durante o mesmo período. Os pacientes previamente implantados com TV-CDI de câmara única, que estavam recebendo S-CDI após a remoção do sistema transvenoso devido a uma complicação, formaram seu próprio grupo controle.

O estudo foi aprovado pelo Comitê de Ética do nosso hospital.

### Procedimento de implante do CDI

Em todos os casos, antes de considerar o implante de qualquer um dos dispositivos, foi realizada educação em saúde sobre as consequências físicas e psicológicas que o dispositivo poderia causar em cada paciente.

Foram realizados todos os implantes no laboratório de eletrofisiologia pela mesma equipe médica e de enfermagem.

Antes do implante, todos os pacientes receberam antibióticos intravenosos profiláticos. O implante ocorreu sem a retirada de medicação anticoagulante oral, exceto nos casos de baixo risco tromboembólico (CHADS-VASc < 2).

Os parâmetros hemodinâmicos (pressão arterial, frequência cardíaca e saturação arterial de oxigênio) foram monitorados de forma não invasiva durante o procedimento.

### Procedimento de implante do CDI subcutâneo

Todos os pacientes foram aprovados no teste de triagem de ECG pré-implante de CDI em pelo menos uma derivação na região paraesternal direita ou esquerda. O procedimento ocorreu sob anestesia local e sedação, de acordo com um protocolo de sedação consciente ( Tabela 1 ). Este foi adaptado de um protocolo de sedação ^20^ previamente descrito, que é usado rotineiramente em nosso hospital para realizar procedimentos intervencionistas complexos.

**Tabela 1:** Protocolo para sedação consciente durante o implante de S-CDI

Pré-medicação: Administrado na chegada ao laboratório de eletrofisiologia, enquanto o paciente está sendo monitorado:
**Ondansetron 8 mg**
**Paracetamol 1 g**
**Midazolam 1 mg**
**Petidina 25 mg**
**Bomba de infusão contínua: 0,30 mg fentanil (2 ampolas) + 120 cc solução salina fisiológica**
Se < 65 kg: 30 ml/h
Se > 65 kg: 40 ml/h
**Durante o procedimento:**
Doses únicas de midazolam ou fentanil em bolus sob demanda
**Medicação do teste de desfibrilação (com a bomba de infusão de fentanil parada):**
3-5 mg bolus de etomidato
3-5 mg bolus de midazolam
**Mediação de resgate preparada:**
Atropina
Naloxona
Flumazenil

A técnica de implante do S-CDI foi conforme descrito anteriormente. ^21^ Em todos os casos, o gerador foi inserido no quinto ou sexto espaço intercostal esquerdo, e o eletrodo de desfibrilação foi posicionado na região paraesternal direita ou esquerda, dependendo dos resultados ou achados do teste de triagem durante o procedimento de implante. Foi utilizada a técnica de duas incisões em todos os casos. ^22^

Ao final do procedimento, foi realizado teste de desfibrilação e foram programadas duas zonas de choque com frequência cardíaca mínima de 200 bpm.

### Procedimento de implante do CDI transvenoso

O implante foi realizado sob anestesia local e leve sedação sob demanda. Através da veia subclávia esquerda, um eletrodo de desfibrilação de bobina única de fixação ativa foi conectado ao ápice do ventrículo direito. O gerador foi inserido por via subcutânea na região infraclavicular esquerda. Nenhum paciente foi submetido a teste de desfibrilação. Os dispositivos foram programados no modo VVI com frequência cardíaca mínima de 40 bpm. A programação terapêutica dos dispositivos foi feita de forma individualizada, de acordo com a indicação do implante de CDI e o tipo de doença cardíaca.

### Acompanhamento

O acompanhamento consistiu em visitas ao local após 15 dias, 3 meses e, em seguida, a cada 6 meses após o implante. Foram registradas as complicações intra-operatórias, peri-operatórias e de longo prazo, assim como a ocorrência de terapia apropriada ou inadequada.

### Questionários sobre qualidade de vida e satisfação/desconforto com o tipo de sistema

Pelo menos 3 meses após o implante do sistema, foi realizada uma pesquisa por telefone. Isso incluiu dois questionários: 1) o questionário de saúde de 12 itens, versão abreviada (SF-12, sigla em inglês) e 2) um questionário especificamente elaborado para comparar a gravidade de dor/desconforto relacionada ao tipo de sistema e à técnica cirúrgica (ICD QoL, sigla em inglês) (Materiais Suplementares 1 e 2).

Os questionários foram aplicados por telefone pelo mesmo investigador, que era cego ao tipo de sistema implantado.

#### SF-12

O questionário SF-12 consiste em um subconjunto de 12 itens do SF-36, selecionados por meio de regressão múltipla. Os resumos dos componentes físicos e mentais da qualidade de vida dos pacientes foram elaborados com base nesses itens.

As opções de resposta do SF-12 estão na forma de escalas de Likert que avaliam a intensidade ou a frequência. O número de opções de resposta varia de 3 a 6, dependendo do item, e cada pergunta recebe um valor que é posteriormente transformado em uma escala de 0 a 100. As pontuações têm uma média de 50 com um desvio padrão de 10. Portanto, os valores acima ou abaixo de 50 indicam melhor ou pior estado de saúde, respectivamente, do que a população de referência. Estudos publicados sobre as características da medição do SF-12 indicam a sua confiabilidade, validade e sensibilidade (alfa de Cronbach > 0,7; coeficiente de correlação intraclasse para reprodutibilidade teste-reteste rho ≥ 0,75). ^23 - 25^

#### ICD QoL

O questionário ICD QoL consistiu-se de 8 itens que avaliam a intensidade da dor (intra-, peri- e pós-procedimento e dor de longa duração), grau de desconforto estético, limitações às atividades de vida diária e de lazer, limitações físicas do sono devido ao potencial desconforto causado pela compressão mecânica pelo dispositivo e satisfação do paciente. Todos os parâmetros do questionário foram medidos em uma escala numérica de classificação de gravidade de 0 a 10. A dor foi definida da maneira seguinte: dor intra-procedimento como dor sofrida durante a intervenção; dor peri-operatória como dor ocorrida durante a internação; dor pós-procedimento como dor dentro de 3 meses após a alta; e dor de longa duração como dor de 3 meses após o implante até o momento da pesquisa. A intensidade da dor foi medida por meio da escala de avaliação numérica, em que 0 significa “sem dor” e 10 significa “pior dor imaginável”. ^26^

Os sete pacientes com S-CDI que também tiveram um TV-CDI no passado responderam ao questionário para ambos os tipos de CDI. Esses pacientes também foram questionados sobre qual dos dois tipos de CDI eles recomendariam.

## Análise estatística

As variáveis contínuas são expressas por meio de estatísticas de tendência central e *spread* (média e desvio padrão para variáveis normalmente distribuídas; mediana e intervalo interquartil para variáveis não paramétricas). Os testes de normalidade foram realizados com o teste de Lilliefors (Kolmogorov-Smirnov). As variáveis categóricas são expressas como porcentagens.

Para comparar as características gerais de ambos os grupos, usamos o teste do qui-quadrado para variáveis qualitativas dicotômicas, o teste t de Student para amostras independentes para variáveis quantitativas paramétricas (assumindo variâncias iguais em todos os casos porque o teste de Levene foi > 0,05) e o teste U de Mann-Whitney para variáveis não paramétricas. Significância estatística foi definida como p < 0,05.

Foi utilizado o teste U de Mann-Whitney para comparar os resultados do questionário SF-12, enquanto os resultados do ICD QoL foram comparados por meio do teste do qui-quadrado.

Foram realizados os cálculos com o pacote estatístico SPSS (Versão 19, SPSS Inc., Chicago, IL, EUA).

## Resultados

### Características de linha de base

Foram inscritos 71 pacientes com CDI. As suas características são apresentadas na Tabela 2 . Em total, 64 pacientes foram submetidos ao primeiro implante de S-CDI ou TV-CDI. Nos outros 7 pacientes, um S-CDI foi implantado após a remoção de um TV-CDI. Os motivos da remoção do sistema transvenoso foram endocardite, infecção da bolsa, úlcera por pressão e deslocamento do eletrodo ( Tabela 3 ). Não foram encontradas diferenças significativas nas características de linha de base dos pacientes de acordo com o tipo de sistema implantado. A média de idade foi de 53 anos (mínimo 13; máximo 76) e 80% dos pacientes eram do sexo masculino. A doença cardíaca de base mais comum foi doença isquêmica (37%), seguida de cardiomiopatia hipertrófica (20%). Na maioria dos casos (56%), o sistema foi implantado como prevenção primária de MSC.

**Tabela 2:** – Características de linha de base dos pacientes

	Geral N=71	TV-CDI	S-CDI	Valor p
**Idade (anos)**	53 ± 11 ^a^	52 ± 10,3 ^a^	50,8 ± 10,6 ^a^	p=0,869 ^b^
**Sexo masculino (%)**	62 (79,5)	31 (80)	31 (80)	p=1 ^c^
**Índice de massa corporal**	24,8 ± 4,6 ^a^	25,8 ± 3,7 ^a^	25,6 ± 4,3 ^a^	p=0,876 ^b^
**Tipo de prevenção (%)**				p=0,648 ^c^
Primário	44 (56,4)	23 (59)	21 (53,8)	
Secundário	34 (43,6)	16 (41)	18 (46,2)	
**Tipo de doença cardíaca (%)**				p=0,319 ^c^
Isquêmica	29 (37,2)	14 (35,9)	15 (38,5)	p=0,319 ^c^
Valvular	2 (2,6)	1 (2,6)	1 (2,6)	p=0,319 ^c^
Dilatada idiopática	6 (7,7)	4 (10,3)	2 (5,1)	p=0,319 ^c^
Hipertrófica	16 (20,5)	5 (12,8)	11 (28,2)	p=0,319 ^c^
Não compactada	6 (7,7)	3 (7,7)	3 (7,7)	p=0,319 ^c^
Brugada	2 (2,6)	1 (2,6)	1 (2,6)	p=0,319 ^c^
QT prolongado	6 (7,7)	2 (5,1)	4 (10,3)	p=0,319 ^c^
Congênita	5 (6,4)	5 (12,8)	0	p=0,319 ^c^
Desconhecida	6 (7,7)	4 (10,3)	2 (5,1)	p=0,319 ^c^
**Fração de ejeção (%)**	46 ± 30 ^a^	45,8 ± 14,8 ^a^	44,8 ± 16 ^a^	p=0,867 ^b^
**Ritmo durante implante (%)**				p=0,867 ^c^
Sinusal	70 (90)	35 (89,7)	35 (89,7)	
Fibrilação atrial	8 (10)	4 (10,3)	4 (10,3)	
**Tx antiplaquetária (%)**	31 (39,7)	15 (38,5)	16 (41)	p=0,817 ^c^
**Tx anticoagulante (%)**	14 (19,7)	7 (17,9)	7 (17,9)	p=1 ^c^

*Tx: terapia;* ^*a*^ *Média e desvio padrão;* ^*b*^ *Teste d de Student para amostras independentes;* ^*c*^ *Teste do qui-quadrado.*

**Tabela 3:** – Motivos para remoção do TV-CDI

Motivos para substituição	N (%)
Endocardite	2 (28,6)
Infecção de bolsa recorrente	2 (28,6)
Fratura de eletrodo	2 (28,6)
Decúbito de bolsa	1 (14,3)

### Acompanhamento

Os resultados do acompanhamento dos pacientes estão resumidos na Tabela 4 . Em termos de complicações peri-operatórias, um paciente do grupo S-CDI apresentou hematoma de bolsa que exigiu drenagem cirúrgica. Um paciente do grupo TV-CDI apresentou deslocamento do eletrodo como complicação durante o acompanhamento.

**Tabela 4:** – Acompanhamento dos pacientes

Complicações	Geral	TV-CDI	S-CDI	Valor p
Complicações peri-operatórias (%)	1 (1,3)	0	1 (2,6)	p=0,314 ^a^
Pneumotórax	0	0	0	
Derrame pericárdico	0	0	0	
Hematoma de bolsa	1 (1,3)	0	1 (2,6)	p=0,152 ^a^
Complicações durante acompanhamento (%)	1 (1,3)	1 (2,6)	0	p=0,152 ^a^
Infecção de bolsa	0	0	0	
Endocardite infecciosa	0	0	0	
Trombose venosa	0	0	0	
Deslocamento de eletrodo	1 (1,3)	1 (2,6)	0	p=0,314 ^a^
Decúbito de bolsa	0	0	0	
Terapia				
Terapia apropriada (%)	11 (14,1)	9 (23,1)	2 (5,1)	p=0,023
ATP	2 (2,6)	2 (5,1)	0	
Choque	9 (11,5)	7 (17,9)	2 (5,1)	
Terapia inapropriada (%)	2 (2,6)	1 (2,6)	1 (2,6)	p=1 ^a^

*ATP: estimulação de antitaquicardia. ^a^ Teste do qui-quadrado.*

Dois pacientes com S-CDI e 9 com TV-CDI receberam terapia apropriada (2 casos foram tratados com estimulação de antitaquicardia e choque elétrico foi necessário em 7 casos). Um paciente com TV-CDI recebeu terapia inadequada por causa do deslocamento do eletrodo ventricular. Outro paciente, com S-CDI, sofreu um choque inadequado devido a taquicardia supraventricular com frequência cardíaca acima do nível de corte terapêutico (240 bpm).

### Questionários

A Tabela 5 mostra os resultados obtidos do questionário ICD QoL em pacientes implantados pela primeira vez. Não foram encontradas diferenças significativas nas avaliações em relação à dor intra-operatória de acordo com o tipo de sistema implantado. No entanto, os pacientes implantados com S-CDI apresentaram dor peri-operatória mais intensa. Não foram encontradas diferenças significativas entre os dois tipos de sistema em termos de distúrbios do sono, embora tenha havido uma tendência de sono mais perturbado entre os receptores de S-CDI. Na maioria dos pacientes, esses distúrbios foram de gravidade baixa a moderada. Da mesma forma, não houve diferenças significativas nas atividades diárias ou no desconforto estético. Todos os pacientes, independentemente do sistema implantado, estavam satisfeitos com a intervenção e disseram que recomendariam o dispositivo a outros pacientes elegíveis.

**Tabela 5:** – Resultados do questionário ICD QoL em pacientes implantados com primeiro CDI

	Subcutâneo N=32	Transvenoso N=32	Valor p
**Dor intra-operatória**			p=0,073 ^a^
Nenhuma dor	23 (74,2)	21 (65,6)	
Leve	5 (16,1)	3 (9,4)	
Moderada	0	5 (15,6)	
Intensa	3 (9,7)	1 (3,1)	
Muito intensa	0	2 (6,3)	
**Dor peri-operatória**			p=0,005 ^a^
Nenhuma dor	9 (29)	15 (46,9)	
Leve	5 (16,1)	12 (37,5)	
Moderada	7 (22,6)	5 (15,6)	
Intensa	9 (29)	0	
Muito intensa	1 (3,2)	0	
**Dor pós-operatória**			p=0,170 ^a^
Nenhuma dor	13 (41,9)	22 (68,8)	
Leve	10 (32,3)	6 (18,8)	
Moderada	5 (16,1)	4 (12,5)	
Intensa	1 (3,2)	0	
Muito intensa	2 (6,5)	0	
**Dor atual**			p=0,087 ^a^
Nenhuma dor	27 (87,1)	26 (81,3)	
Leve	1 (3,2)	6 (18,8)	
Moderada	2 (6,5)	0	
Intensa	0	0	
Muito intensa	1 (3,2)	0	
**Desconforto estético**			p=0,683 ^a^
Nenhum	20 (64,5)	21 (65,6)	
Leve	7 (22,6)	6 (18,8)	
Moderado	3 (9,7)	2 (6,3)	
Muito	0	2 (6,3)	
Extremo	1 (3,2)	1 (3,1)	
**Atividades da vida diária limitadas**			p=0,080 ^a^
Nenhuma	22 (71)	22 (68,8)	
Poucas	1 (3,2)	7 (21,9)	
Moderadas	5 (16,1)	2 (6,3)	
Muitas	3 (9,7)	1 (3,1)	
Extremas	0	0	
**Distúrbio do sono**			p=0,232 ^a^
Nenhum	13 (41,9)	21 (65,6)	
Leve	10 (32,3)	8 (25)	
Moderado	5 (16,1)	3 (9,4)	
Grave	2 (6,5)	0	
Muito grave	1 (3,2)	0	
**Recomendaria para outros**			
Sim	31 (100)	32 (100)	
Não	0	0	
**Satisfeito com a intervenção**			
Sim	31 (100)	32 (100)	
Não	0	0	

*^a^ Teste do qui-quadrado.*

Os resultados obtidos com o questionário SF-12 são apresentados na Tabela 6 . Valores semelhantes foram registrados em ambos os grupos, com medianas de 44,3 ± 12,8 para o grupo S-CDI e 48,8 ± 9,8 para o grupo TV-CDI na escala de saúde física. A escala de saúde mental apresentou medianas de 45,9 ± 13,7 para o S-CDI e 50,8 ± 10,3 para o TV-CDI.

**Tabela 6:** – Resultados do questionário SF-12 em pacientes implantados com primeiro CDI

	Subcutâneo N=32				Transvenoso N=32				Valor p

	Mediana	IIQ	Mínimo	Máximo	Mediana	IIQ	Mínimo	Máximo	
Escala de saúde física	44,3	12,8	27,4	56,7	48,8	9,8	31,6	62,6	p=0,302 ^a^
Escala de saúde mental	45,9	13,7	26,3	56,8	50,8	10,3	18,7	55,5	p=0,345 ^a^
Escala de função física	47,9	17,2	22,1	56,5	56,5	15,1	22,1	56,5	p=0,099 ^a^
Escala de limitação física	29,5	9,2	20,3	29,5	29,5	9,2	20,3	29,5	p=0,656 ^a^
Escala de dor	57,4	0	16,7	57,4	57,4	0	37,1	57,4	p=0,150 ^a^
Escala de saúde geral	44,7	10,8	18,9	62	55,5	10,8	18,9	62	p=0,354 ^a^
Escala de vitalidade	57,8	30,2	17,6	67,9	67,9	20,2	27,6	67,9	p=0,157 ^a^
Escala de limitação emocional	56,6	9,2	16,2	56,6	56,6	10,1	26,3	56,6	p=0,317 ^a^
Escala de função social	22,5	0	11,3	22,5	22,5	0	11,3	22,5	p=0,263 ^a^
Escala de saúde mental 2	64,5	18,3	21,9	70,6	64,5	18,3	21,9	70,6	p=0,163 ^a^

*IIQ: Intervalo interquartil. ^a^ Teste U de Mann-Whitney.*

As Tabelas 7 e 8 mostram os resultados dos questionários ICD QoL e SF-12, respectivamente, em pacientes implantados com S-CDI após a remoção de um sistema transvenoso. Em termos de avaliações da dor intra-operatória, nenhum paciente no grupo S-CDI relatou dor, em comparação com 57% que relataram dor com os sistemas transvenosos. Essa dor foi moderadamente forte, no máximo. Não foram encontradas diferenças estatisticamente significativas na dor peri-operatória, pós-operatória ou de longo prazo. Da mesma forma, não houve diferenças entre os tipos de CDI em relação aos distúrbios do sono ou ao grau de desconforto estético. Todos os pacientes ficaram satisfeitos com a intervenção e recomendariam a implantação do dispositivo, se necessário. Quando questionados sobre qual tipo de CDI eles recomendariam, todos, exceto um, preferiram o sistema subcutâneo.

**Tabela 7:** – Resultados do questionário ICD QoL em pacientes implantados com S-CDI após a remoção de um TV-CDI

	Subcutâneo N=7	Transvenoso N=7	Valor p
**Dor intra-operatória**			P=1 ^a^
Nenhuma dor	7 (100)	3 (42,9)	
Leve	0	2 (28,6)	
Moderada	0	2 (28,6)	
Intensa	0	0	
Muito intensa	0	0	
**Dor peri-operatória**			p=0,224 ^a^
Nenhuma dor	4 (57,1)	4 (57,1)	
Leve	2 (28,6)	1 (14,3)	
Moderada	1 (14,3)	2 (28,6)	
Intensa	0	0	
Muito intensa	0	0	
**Dor pós-operatória**			p=0,659 ^a^
Nenhuma dor	6 (87,1)	6 (87,1)	
Leve	1 (14,3)	1 (14,3)	
Moderada	0	0	
Intensa	0	0	
Muito intensa	0	0	
**Dor atual**			p=0,659 ^a^
Nenhuma dor	6 (87,1)	6 (87,1)	
Leve	1 (14,3)	1 (14,3)	
Moderada	0	0	
Intensa	0	0	
Muito intensa	0	0	
**Desconforto estético**			p=0,717 ^a^
Nenhum	5 (71,4)	4 (57,1)	
Leve	1 (14,3)	2 (28,6)	
Moderado	1 (14,3)	1 (14,3)	
Muito	0	0	
Extremo	0	0	
**Atividades da vida diária limitadas**			p=0,427 ^a^
Nenhuma	5 (71,4)	5 (71,4)	
Poucas	2 (28,6)	2 (28,6)	
Moderadas	0	0	
Muitas	0	0	
Extremas	0	0	
**Distúrbio do sono**			p=0,350 ^a^
Nenhum	5 (71,4)	4 (57,1)	
Leve	0	0	
Moderado	1 (14,3)	3 (42,9)	
Grave	1 (14,3)	0	
Muito grave	0	0	

*^a^ Teste do qui-quadrado.*

**Tabela 8:** – Resultados do questionário SF-12 em pacientes implantados com S-CDI após a remoção de um TV-CDI

	Mediana	IIQ	Mínimo	Máximo
Escala de saúde física	51,3	5,3	30,5	52,9
Escala de saúde mental	46	4,8	40,2	51,3
Escala de função física	56,5	0	22,1	56,5
Escala de limitação física	29,5	0	20,3	29,5
Escala de dor	57,4	10,1	47,3	57,4
Escala de saúde geral	55,5	10,8	29,6	62
Escala de vitalidade	57,8	0	27,6	67,9
Escala de limitação emocional	56,6	10,1	16,2	56,6
Escala de função social	22,5	0	11,3	22,5
Escala de saúde mental 2	58,4	6,1	58,4	64,5

*IIQ: Intervalo interquartil.*

## Discussão

Este estudo demonstra que não há diferenças estatisticamente significativas no impacto na qualidade de vida em pacientes com S-CDI versus aqueles com TV-CDI. Além disso, a avaliação específica de variáveis que se mostram mais controversas na avaliação e escolha do tipo de sistema a ser implantado, como parâmetros relacionados ao procedimento cirúrgico ou especificações técnicas do dispositivo, também não apresentaram diferenças significativas entre os dois grupos de pacientes.

Os resultados de estudos prévios sobre o impacto do CDI na qualidade de vida dos pacientes são contraditórios. Enquanto alguns estudos verificaram que a qualidade de vida piorou ou não mudou significativamente após o implante de CDI, ^27^ outros notaram melhora gradual. ^28^ No entanto, apenas um estudo até o momento presente avaliou a qualidade de vida em pacientes com S-CDI. O EFFORTLESS QoL ^19^ é um subestudo de registro multicêntrico internacional que comparou a qualidade de vida em pacientes com S-CDI com uma população histórica de pacientes com TV-CDI. Não foram encontradas diferenças estatisticamente significativas na qualidade de vida, conforme avaliada pelo questionário SF-12.

Os resultados do nosso estudo assemelham aos de Pedersen et al., ^19^ Nossos resultados do questionário de qualidade de vida SF-12, administrado a pacientes implantados com CDI pela primeira vez, não mostraram diferenças nas escalas de saúde mental ou física.

O nosso é o primeiro estudo a avaliar o impacto na qualidade de vida em pacientes com S-CDI, enfatizando a análise de características potenciais (envolvendo tanto a técnica cirúrgica quanto o tipo de sistema implantado) que podem influenciar os resultados. Muitos estudos já demonstraram a eficácia e segurança desse tipo de CDI em comparação com os dispositivos convencionais. Isso permitiu que as indicações fossem ampliadas e contribuiu para a aprovação da equipe médica. Ainda hoje, no entanto, algumas incertezas são frequentemente encontradas entre os pacientes e, especialmente, entre os profissionais de saúde, na hora de indicar e escolher esse tipo de sistema em pacientes selecionados, principalmente em função da diferença de tamanho, da localização diferente e da técnica de implante. Na tentativa de abordar essas questões, elaboramos um questionário específico e comparamos a nossa população de pacientes com S-CDI com um grupo de TV-CDI sem indicação para terapia de antibradicardia, estimulação de antitaquicardia ou terapia de ressincronização (i.e., potenciais candidatos para S-CDI). Os pacientes foram pareados por idade, sexo e índice de massa corporal. Nós consideramos estas como potenciais variáveis de confusão ao avaliar o impacto na qualidade de vida de acordo com o tipo de sistema implantado.

É evidente que algum grau de dor ocorreu em geral com ambos os sistemas, a dor peri-operatória sendo mais intensa entre os pacientes com S-CDI. Não houve diferenças na intensidade da dor intra-operatória ou de longo prazo. O fato do manejo pós-operatório ter sido um pouco inconsistente nesses pacientes pode ter influenciado este resultado, uma vez que esses pacientes são internados na enfermaria e atendidos por diferentes equipes médicas e de enfermagem. No entanto, estes achados são indubitavelmente relevantes, e os receptores de S-CDI devem, portanto, receber analgesia perioperatória mais forte. Não foram encontradas diferenças estatisticamente significativas quando o desconforto estético, os distúrbios do sono e as atividades diárias foram comparados entre os dois grupos.

Outro aspecto inovador deste estudo é a avaliação da percepção da qualidade de vida em pacientes que fizeram os dois tipos de terapia. Estes pacientes relataram dor intra-operatória, desconforto estético e distúrbios do sono mais graves com o sistema transvenoso, embora essas diferenças não sejam estatisticamente significativas, possivelmente devido ao pequeno tamanho da amostra do grupo (7 pacientes). Esta foi, inegavelmente, uma população tendenciosa, pois o sistema subcutâneo foi implantado após a ocorrência de uma complicação do sistema transvenoso. Os parâmetros avaliados, no entanto, como gravidade da dor durante a intervenção cirúrgica, distúrbios do sono e desconforto estético, não estão relacionados às complicações que surgiram com o dispositivo convencional. Portanto, essas questões são potencialmente independentes das repercussões negativas desse sistema.

Estes dados demonstram que o tamanho e localização diferentes do S-CDI não influenciam a qualidade de vida dos pacientes negativamente.

Por outro lado, nosso estudo fornece os primeiros dados sobre segurança do paciente e conforto/dor durante intervenções cirúrgicas para implante de um S-CDI utilizando um protocolo de sedação consciente, que é completamente administrado por uma equipe de eletrofisiologia (equipe médica e de enfermagem). Embora o TV-CDI seja atualmente implantado principalmente sob anestesia local, o S-CDI é implantado sob anestesia geral na maioria dos hospitais. No maior estudo multicêntrico até o momento, 63% dos locais implantaram S-CDI sob anestesia geral. ^5^ Esse recurso tem disponibilidade limitada na maioria dos locais. Envolve esforço organizacional, mais pessoal durante a intervenção e maiores custos de saúde. A literatura contém várias séries de casos clínicos que descrevem experiências com o implante de S-CDI sob sedação, com supervisão estrita por anestesistas especialistas. O estudo de Essandoh et al., ^29^ retrospectivamente analisou a eficácia e a segurança do implante de S-CDI sob sedação supervisionada por um anestesiologista, em um total de 10 pacientes selecionados. Não foram relatadas complicações hemodinâmicas ou respiratórias.

A segurança e a eficácia da sedação consciente já foram demonstradas em pacientes submetidos à ablação por fibrilação atrial, ^20^ e este método é usado rotineiramente em nosso laboratório. Para o implante de S-CDI, utilizamos um protocolo de sedação adaptado para esse tipo de procedimento, a fim de garantir analgesia adequada para os pacientes durante toda a intervenção. Nenhuma complicação foi registrada durante o procedimento. Deve-se notar que 100% dos pacientes implantados com ambos os tipos de sistema descreveram uma ausência completa de dor durante o implante de S-CDI, enquanto menos da metade desses pacientes relataram não ter sentido qualquer dor durante o procedimento de TV-ICD.

### Limitações

Uma limitação do estudo seria o potencial viés do entrevistador. Para evitar isso, as pesquisas foram administradas por telefone pelo mesmo investigador cego. Para evitar viés de memória nos sujeitos entrevistados, foram incluídos apenas pacientes implantados com CDI nos últimos 2 anos.

A população de controle consistia em pacientes com TV-CDI pareados por idade, sexo e índice de massa corporal. Acreditamos que estas variáveis possam influenciar a resposta dos pacientes em relação ao grau de desconforto/satisfação com S-CDI versus TV-CDI. No entanto, outras variáveis não controladas pelo desenho do estudo, como indicação de CDI, tipo de doença cardíaca ou classe funcional, bem como a qualidade de vida pré-implantação, podem ter influenciado a qualidade de vida desses pacientes, afetando a avaliação do impacto específico do CDI. Porém, a ausência de diferenças estatisticamente significativas nas características de linha de base dos pacientes diminui consideravelmente essa limitação potencial.

Uma possível limitação deste estudo é a menor prevalência de choques sofridos pelo grupo S-CDI (5,1% versus 17,9%), o que poderia ter alguma influência na percepção da qualidade de vida ao analisar este subgrupo de pacientes. No entanto, a prevalência de choques foi baixa em ambos os grupos (11%). Portanto, acreditamos que isso não influenciou significativamente os resultados gerais de nosso estudo.

Por último, o tamanho da amostra foi pequeno, sendo obtida em apenas um hospital, assim limitando o poder estatístico necessário para detectar diferenças. No entanto, nossos dados de qualidade de vida se assemelham aos publicados recentemente em uma população maior. ^5^

## Conclusões

O tipo de CDI implantado não influencia significativamente a percepção dos pacientes sobre a qualidade de vida física ou mental. Nosso estudo demonstra que as diferenças no procedimento cirúrgico (local e técnica cirúrgica) ou o tipo de sistema implantado (por exemplo, peso e tamanho) não têm impacto negativo na qualidade de vida do paciente. Por outro lado, esses achados sugerem que o S-CDI pode ser implantado com segurança sob sedação consciente por uma equipe de eletrofisiologia. Estudos maiores e randomizados são necessários para comparar e confirmar estes resultados.

### Pontos chaves

#### O que já se sabe sobre esse assunto?

- O CDI subcutâneo demonstrou ser semelhante em eficácia ao CDI convencional na prevenção da morte súbita cardíaca.
- O CDI subcutâneo é uma alternativa ao CDI transvenoso em pacientes que não requerem antibradicardia, antitaquicardia ou estimulação de ressincronização cardíaca; pacientes com difícil acesso venoso; pacientes jovens; ou após a remoção de um CDI convencional devido à infecção.
- O CDI subcutâneo emprega uma técnica cirúrgica diferente do CDI convencional e o gerador é maior e mais pesado do que nos sistemas transvenosos atuais.

#### O que este estudo acrescenta?

- Não há diferenças significativas na qualidade de vida física ou mental entre uma população espanhola de pacientes com CDI subcutâneo ou transvenoso.
- Diferenças na técnica cirúrgica ou no tipo de sistema implantado não afetam negativamente a qualidade de vida do paciente.
- Pacientes implantados com CDI subcutâneo após a remoção de um CDI transvenoso devido a complicações avaliam o novo dispositivo positivamente.
- O CDI subcutâneo pode ser implantado com segurança sob sedação consciente por uma equipe de eletrofisiologia.

## * Material suplementar

Para informação adicional, por favor, clique aqui.
