# Supplementary material for: Estudo Comparativo entre Receptores de Desfibriladores Subcutâneos e Transvenosos em Relação à Tolerância ao Procedimento de Implante e Percepção da Qualidade de Vida
Source: Arq Bras Cardiol. 2021 Jun 8;116(6):1139–48. [Article in Portuguese] doi: 10.36660/abc.20190312 (PMC8288548; doi:10.36660/abc.20190312)
Supplement: Supplementary file 1 [file 2019-0312-supplementary-material.pdf]

Anexo 3. Cuestionario sobre dolor, limitaciones en las actividades cotidianas y disconfort estético (QL DAI).

Código de paciente:

Dolor, registrar las respuestas de cada pregunta en la tabla correspondiente a continuación.

1. En cuanto al dolor durante el implante del dispositivo, que valor en intensidad le otorgaría en una escala del 1 al 5, siendo uno nada y 5 muchisimo.
2. En cuanto al dolor en la región del DAI, el dia del implante del dispositivo, que valor en intensidad le otorgaría en una escala del 1 al 5, siendo uno nada y 5 muchisimo.
3. En cuanto al dolor dolor en la región del DAI, en los primeros 3 meses post implante del dispositivo, que valor en intensidad le otorgaría en una escala del 1 al 5, siendo uno nada y 5 muchisimo.
4. En cuanto al dolor en la región del DAI, pasados los primeros 3 meses hasta la actualidad , que valor en intensidad le otorgaría en una escala del 1 al 5, siendo uno nada y 5 muchisimo.
5. En cuanto a las limitaciones producidas por el dispositivo en sus actividades cotidianas de la vida diaria, que valor le daría usted en una escala del 1 al 5.
6. En cuanto a las limitaciones producidas por el dispositivo para el descanso nocturno, usted que valor le daría en una escala del 1 al 5.

Tabla para registrar respuestas de la encuesta sobre dolor y limitaciones

Variable	1	2	3	4	5
Dolor intraprocedimiento					
Dolor periprocedimiento					
Dolor primeros 3 meses					
Dolor > 3meses					
Limitación actividades cotidianas					
Limitación al dormir					

Recomendaría usted el dispositivo a una persona que lo necesita:

SI NO

Esta usted satisfecho con todo el proceso de llevar el dispositivo

SI NO

Firma y nombre de medico:

Fecha:
