# Supplementary material for: Estudo Comparativo entre Receptores de Desfibriladores Subcutâneos e Transvenosos em Relação à Tolerância ao Procedimento de Implante e Percepção da Qualidade de Vida
Source: Arq Bras Cardiol. 2021 Jun 8;116(6):1139–48. [Article in Portuguese] doi: 10.36660/abc.20190312 (PMC8288548; doi:10.36660/abc.20190312)
Supplement: Translation

# Translation: Comparative Study between Subcutaneous and Endovascular Defibrillator Recipients Regarding Tolerance to the Implant Procedure and Perception of Quality of Life

**Authors:** Pablo Esteban Auquilla-Clavijo, Naiara Calvo-Galiano, Marina Povar-Echeverría, Teresa Oloriz-Sanjuan, Francisco Diaz-Cortejana, Antonio Asso-Abadia

**Affiliations:** 1 Espanha Hospital Universitario Miguel Servet – Cardiologia, Zaragoza - Espanha; 2 Espanha Hospital Universitario Miguel Servet-Medicina Interna, Zaragoza - Espanha

**Keywords:** Defibrilators, Implantable, Defibrillators Subcutaneous Implantable, Comparative Study, Conscious Sedation, Quality of Life

## Abstract

**Background:**

The totally subcutaneous implantable cardioverter-defibrillator (S-ICD) is a safe alternative to the conventional transvenous ICD (TV-ICD) system to prevent sudden death.

**Objective:**

To compare the impact of the type of ICD system and surgical technique on patients’ quality of life, as well as the severity of discomfort and pain, between S-ICD and TV-ICD recipients.

**Methods:**

Consecutively implanted patients with an S-ICD system were matched with patients with a TV-ICD system. In addition, patients undergoing S-ICD implantation after removal of a TV-ICD due to complications were included. Quality of life (measured with the 12-item short-form health survey) and severity of pain and discomfort were evaluated. Statistical significance was defined as p < 0.05.

**Results:**

A total of 64 patients implanted with S-ICD or TV-ICD under local anesthesia and conscious sedation were analyzed. Patients with S-ICD and TV-ICD systems did not differ significantly in quality of life scores. S-ICD patients had a higher level of perioperative pain; no differences were found regarding severity of intraoperative pain. The magnitude of aesthetic discomfort and sleep disturbances did not differ between groups. An S-ICD was implanted in 7 additional patients after removal of a TV-ICD. All but one of these patients recommended the S-ICD system.

**Conclusions:**

The type of ICD system and the surgical technique have negligible impact on patients’ quality of life. These results suggest that conscious sedation, provided by an experienced electrophysiology team, could be considered as an alternative to general anesthesia to manage patients undergoing S-ICD implantation.

## Introduction

Sudden cardiac death (SCD) of arrhythmic origin is the main cause of cardiovascular mortality. The efficacy of the implantable cardioverter-defibrillator (ICD) for reducing SCD mortality in selected populations has been extensively demonstrated in many clinical trials.^1^ Conventional defibrillator systems consist of a pulse generator located in the pectoral area, connected to the endocardium by means of transvenous leads. This type of device is therefore prone to complications inherent in the mechanism of implantation and the intravascular position of the leads.

Because of problems accessing the heart through the venous system and the potential for complications, the subcutaneous implantable cardioverter-defibrillator (S-ICD, Boston Scientific, Natick, MA, USA) has been developed. This system consists of a generator (S-ICD^®^, EMBLEM MRI S-ICD A219, Boston Scientific) connected to a lead (3401, Boston Scientific) located subcutaneously in a parasternal position, generally on the left.^2^ Current clinical guidelines include it with a Class IIa indication as an alternative to the conventional transvenous ICD (TV-ICD) in patients who do not require antibradycardia, antitachycardia, or resynchronisation therapy. It also has a Class IIb indication in patients with no venous access, following removal of a transvenous system due to of infection, and in young patients facing a lifetime requirement for device-based therapy.^2^ A limited implantation-related complication rate has been reported. Moreover, although there are no randomized comparative studies of S-ICDs versus TV-ICDs to date, the available data show the S-ICD to be a very effective device for detecting and treating malignant ventricular arrhythmias.^3 - 6^

The use of S-ICD in Spain is increasingly accepted by scientific societies. The study by Arias et al.^4^ in a Spanish center in 2017 has made it possible to obtain excellent acute and long-term results in a cohort of 50 patients with S-ICD.^7^ The latest Spanish ICD implant registry in 2017 indicates a progressive increase in S-ICD implants from 2.5% in 2015 to 5.3% in 2017.^8^ The higher cost of the S-ICD compared to TV-ICD could be one of the reasons that the adoption of this device has been slow, despite its revolutionary design.^9^ The development of multicentre studies that support these results^7^ would allow us to expand the use of this device.

In terms of the impact of ICD implantation on quality of life, the literature contains contradictory evidence.^10 - 17^ Whereas initial ICD experiences were associated with worse quality of life, more recent studies have demonstrated quality of life at least as good as in patients in the general population, without an ICD.^16 , 18^ The only study to have evaluated and compared quality of life in TV-ICD patients versus S-ICD recipients was published recently. There was an improvement in quality of life in both patient groups, as measured by the SF-12 health survey, and no significant differences were seen between the groups.^19^

## Objective

The objective of our study was to compare perceived quality of life, as well as severity of pain and discomfort, resulting from the surgical technique and type of device, between a population of patients receiving S-ICD and a conventional TV-ICD recipient control group.

## Methods

All patients implanted with an S-ICD at our hospital from 2014 to 2016 were consecutively enrolled. Patients were matched by age, sex, and body mass index with a sample of patients who were undergoing their first implantation of a single-chamber TV-ICD, with no indication for antibradycardia therapy or antitachycardia pacing, during the same period. Patients previously implanted with a single-chamber TV-ICD, who were receiving an S-ICD after having the transvenous system removed due to a complication, formed their own control group.

The study was approved by the Ethics Committee at our hospital.

### ICD implantation procedure

In all cases, prior to considering the implant of either device, health education was carried out regarding the physical and psychological consequences that the device could have on each patient.

All implantations were carried out in the electrophysiology laboratory by the same medical and nursing team.

Prior to implantation, all patients were given prophylactic intravenous antibiotics. Implantation took place without withdrawal of oral anticoagulant medication, except in cases of low thromboembolic risk (CHADS-VASc < 2).

Hemodynamic parameters (blood pressure, heart rate, and arterial oxygen saturation) were non-invasively monitored during the procedure.

### Subcutaneous ICD implantation procedure

All patients passed the ICD pre-implantation ECG screening test in at least one lead in the right or left parasternal region. The procedure took place under local anesthesia and sedation, according to a conscious sedation protocol ( Table 1 ). This was adapted from a previously described sedation protocol^20^ used routinely at our hospital for conducting complex interventional procedures.

**Table 1:** Protocol for conscious sedation during S-ICD implantation

PREMEDICATION: Administered on arrival in the electrophysiology laboratory, once the patient is being monitored:
Ondansetron 8 mg
Paracetamol 1 g
Midazolam 1 mg
Pethidine 25 mg
Continuous infusion pump: 0.30 mg fentanyl (2 ampoules) + 120 cc physiological saline
If < 65 kg: 30 ml/h
If > 65 kg: 40 ml/h
**DURING THE PROCEDURE:**
One-off doses of midazolam or fentanyl as a bolus on demand
**DEFIBRILLATION TEST MEDICATION (with fentanyl infusion pump stopped):**
3-5 mg bolus of etomidate
3-5 mg bolus of midazolam
**PREPARED RESCUE MEDICATION:**
Atropine
Naloxone
Flumazenil

The S-ICD implantation technique was as described previously.^21^ In all cases, the generator was inserted in the fifth or sixth left intercostal space, and the defibrillation lead was positioned in the right or left parasternal region, depending on screening test results or findings during the implantation procedure. The two-incision technique was used in all cases.^22^

At the end of the procedure, a defibrillation test was done, and two shock zones were programmed with a minimum heart rate of 200 bpm.

### Transvenous ICD implantation procedure

Implantation was carried out under local anesthesia and light sedation on demand. Via the left subclavian vein, an active fixation single-coil defibrillation lead was attached to the right ventricular apex. The generator was inserted subcutaneously in the left infraclavicular region. No patients underwent a defibrillation test. The devices were programmed in VVI mode with a minimum heart rate of 40 bpm. Device therapy programming was done on an individual basis, according to the indication for ICD implantation and the type of heart disease.

### Follow-up

Follow-up consisted of site visits after 15 days, 3 months, and then every 6 months post-implantation. Intraoperative, perioperative, and long-term complications were recorded, as was the occurrence of appropriate or inappropriate therapy.

Questionnaires about quality of life and satisfaction/discomfort with the type of system implanted

At least 3 months after the system was implanted, a telephone survey took place. This included two questionnaires: 1) the 12-item short-form health survey (SF-12) and 2) a questionnaire specifically designed to compare the severity of pain/discomfort related to the system type and surgical technique (ICD QoL) (Supplementary Materials 1 and 2).

The survey was administered over the telephone by the same investigator, who was blinded to the type of system implanted.

#### SF-12

The SF-12 survey consists of a subset of 12 items from the SF-36, selected by means of multiple regression. Physical and mental component summaries of patients’ quality of life were designed based on these items.

SF-12 response options take the form of Likert scales evaluating intensity or frequency. The number of response options ranges from 3 to 6, depending on the item, and each question is given a value that is subsequently transformed on a scale of 0 to 100. Scores have a mean of 50 with a standard deviation of 10. Consequently, values above or below 50 indicate a better or worse state of health, respectively, than the reference population. Published studies of SF-12 measurement characteristics indicate reliability, validity, and sensitivity (Cronbach’s alpha > 0.7; intraclass correlation coefficient for test-retest reproducibility rho ≥ 0.75).^23 - 25^

#### ICD QoL

The ICD QoL questionnaire consisted of 8 items evaluating severity of pain (intra-, peri- and post-procedural and long-term pain), degree of aesthetic discomfort, limitations to activities of daily living and leisure activities, physical sleep limitations due to potential discomfort caused by mechanical compression by the device, and patient satisfaction. All parameters in the questionnaire were measured on a numerical severity rating scale from 0 to 10. Pain was defined as follows: intraprocedural pain as pain suffered during the intervention; perioperative pain as pain that occurred during the hospital stay; postprocedural pain as pain within 3 months of being discharged; and long-term pain as pain from 3 months post-implantation up to the time of the survey. Pain severity was measured using the numerical rating scale, where 0 means “no pain” and 10 means “worst pain imaginable”.^26^

The seven S-ICD patients who had also had a TV-ICD in the past answered the questionnaire for both types of ICD. These patients were also asked which of the two types of ICD they would recommend.

## Statistical analysis

Continuous variables are expressed using statistics of central tendency and spread (mean and standard deviation for normally distributed variables; median and interquartile range for non-parametric variables). Normality tests were performed with the Lilliefors (Kolmogorov-Smirnov) test. Categorical variables are expressed as percentages.

To compare the overall characteristics of both groups, we used the chi-square test for dichotomous qualitative variables, Student t test for independent samples for parametric quantitative variables (assuming equal variances in all cases because Levene’s test was > 0.05), and the Mann-Whitney U-test for non-parametric variables. Statistical significance was defined as p < 0.05.

The Mann-Whitney U-test was used to compare the SF-12 survey results, whereas the ICD QoL results were compared by means of the chi-square test.

Calculations were performed with the SPSS statistics package (Version 19, SPSS Inc., Chicago, IL, USA).

## Results

### Baseline characteristics

In all, 71 ICD patients were enrolled. Their characteristics are shown in Table 2 . A total of 64 patients underwent their first implantation of an S-ICD or a TV-ICD. In the other 7 patients, an S-ICD was implanted following removal of a TV-ICD. The reasons for the transvenous system being removed were endocarditis, pocket infection, pressure ulcer, and lead dislodgement ( Table 3 ). No significant differences were found in patient baseline characteristics according to the type of system implanted. Mean age was 53 years (minimum 13; maximum 76), and 80% of patients were male. The most common underlying heart condition was ischemic disease (37%), followed by hypertrophic cardiomyopathy (20%). In most cases (56%), the system was implanted as primary prevention of SCD.

**Table 2:** – Baseline patient characteristics

	Overall N=71	TV-ICD	S-ICD	P value
**Age (years)**	53 ± 11^a^	52 ± 10.3^a^	50.8 ± 10.6^a^	p=0.869^b^
**Male (%)**	62 (79.5)	31 (80)	31 (80)	p=1^c^
**Body mass index**	24.8 ± 4.6^a^	25.8 ± 3.7^a^	25.6 ± 4.3^a^	p=0.876^b^
**Prevention type (%)**				p=0.648^c^
Primary	44 (56.4)	23 (59)	21 (53.8)	
Secondary	34 (43.6)	16 (41)	18 (46.2)	
**Heart disease type (%)**				p=0.319^c^
Ischemic	29 (37.2)	14 (35.9)	15 (38.5)	p=0.319^c^
Valvular	2 (2.6)	1 (2.6)	1 (2.6)	p=0.319^c^
Idiopathic dilated	6 (7.7)	4 (10.3)	2 (5.1)	p=0.319^c^
Hypertrophic	16 (20.5)	5 (12.8)	11 (28.2)	p=0.319^c^
Non-compaction	6 (7.7)	3 (7.7)	3 (7.7)	p=0.319^c^
Brugada	2 (2.6)	1 (2.6)	1 (2.6)	p=0.319^c^
Long QT	6 (7.7)	2 (5.1)	4 (10.3)	p=0.319^c^
Congenital	5 (6.4)	5 (12.8)	0	p=0.319^c^
Unknown	6 (7.7)	4 (10.3)	2 (5.1)	p=0.319^c^
**Ejection fraction (%)**	46 ± 30^a^	45.8 ± 14.8^a^	44.8 ± 16^a^	p=0.867^b^
**Rhythm at implant. (%)**				p=0.867^c^
Sinus	70 (90)	35 (89.7)	35 (89.7)	
Atrial fibrillation	8 (10)	4 (10.3)	4 (10.3)	
**Antiplatelet Tx (%)**	31 (39.7)	15 (38.5)	16 (41)	p=0.817^c^
**Anticoagulant Tx (%)**	14 (19.7)	7 (17.9)	7 (17.9)	p=1^c^

*Tx: therapy; aMean and standard deviation; bStudent t test for independent samples; cChi-square test.*

**Table 3:** – Reasons for TV-ICD removal

Reasons for replacement	N (%)
Endocarditis	2 (28.6)
Recurrent pocket infection	2 (28.6)
Lead fracture	2 (28.6)
Pocket decubitus	1 (14.3)

### Follow-up

Patient follow-up results are summarized in Table 4 . In terms of perioperative complications, one patient in the S-ICD group had a pocket hematoma that required surgical drainage. One TV-ICD patient experienced lead dislodgement as a complication during follow-up.

**Table 4:** – Patient follow-up

Complications	Overall	TV-ICD	S-ICD	P value
Perioperative complications (%)	1 (1.3)	0	1 (2.6)	p=0.314^a^
Pneumothorax	0	0	0	
Pericardial effusion	0	0	0	
Pocket hematoma	1 (1.3)	0	1 (2.6)	p=0.152^a^
Complications during follow-up (%)	1 (1.3)	1 (2.6)	0	p=0.152^a^
Pocket infection	0	0	0	
Infectious endocarditis	0	0	0	
Venous thrombosis	0	0	0	
Lead dislodgement	1 (1.3)	1 (2.6)	0	p=0.314^a^
Pocket decubitus	0	0	0	
Therapy				
Appropriate therapy (%)	11 (14.1)	9 (23.1)	2 (5.1)	p=0.023
ATP	2 (2.6)	2 (5.1)	0	
Shock	9 (11.5)	7 (17.9)	2 (5.1)	
Inappropriate therapy (%)	2 (2.6)	1 (2.6)	1 (2.6)	p=1^a^

*ATP: Antitachycardia pacing.^a^Chi-square test.*

Two patients with S-ICD and 9 with TV-ICD received appropriate therapy (2 cases were treated by antitachycardia pacing, and electric shock was required in 7 cases). One TV-ICD patient received inappropriate therapy because of ventricular lead dislodgement. Another patient, with an S-ICD, suffered an inappropriate shock due to supraventricular tachycardia with a heart rate above the therapy cut-off rate (240 bpm).

### Questionnaires

Table 5 shows the results obtained with the ICD QoL questionnaire in patients implanted for the first time. No significant differences were found with respect to intraoperative pain assessments according to the type of system implanted. However, patients implanted with an S-ICD had more severe perioperative pain. No significant differences were found between the two types of systems in terms of sleep disturbances, although there was a trend towards more disturbed sleep among S-ICD recipients. In most patients, these disturbances were of low to moderate severity. Likewise, there were no significant differences in daily activities or aesthetic discomfort. All patients, regardless of the system implanted, were satisfied with the intervention, and they said they would recommend the device to other eligible patients.

**Table 5:** – ICD QoL questionnaire results in patients implanted with their first ICD

	Subcutaneous N=32	Transvenous N=32	p value
**Intraoperative pain**			p=0.073^a^
No pain	23 (74.2)	21 (65.6)	
Mild	5 (16.1)	3 (9.4)	
Moderate	0	5 (15.6)	
Severe	3 (9.7)	1 (3.1)	
Very severe	0	2 (6.3)	
**Perioperative pain**			p=0.005^a^
No pain	9 (29)	15 (46.9)	
Mild	5 (16.1)	12 (37.5)	
Moderate	7 (22.6)	5 (15.6)	
Severe	9 (29)	0	
Very severe	1 (3.2)	0	
**Postoperative pain**			p=0.170^a^
No pain	13 (41.9)	22 (68.8)	
Mild	10 (32.3)	6 (18.8)	
Moderate	5 (16.1)	4 (12.5)	
Severe	1 (3.2)	0	
Very severe	2 (6.5)	0	
**Current pain**			p=0.087^a^
No pain	27 (87.1)	26 (81.3)	
Mild	1 (3.2)	6 (18.8)	
Moderate	2 (6.5)	0	
Severe	0	0	
Very severe	1 (3.2)	0	
**Aesthetic discomfort**			p=0.683^a^
None	20 (64.5)	21 (65.6)	
Mild	7 (22.6)	6 (18.8)	
Moderate	3 (9.7)	2 (6.3)	
A lot	0	2 (6.3)	
Very much	1 (3.2)	1 (3.1)	
**Activities of daily living limited**			p=0.080^a^
None	22 (71)	22 (68.8)	
A little	1 (3.2)	7 (21.9)	
Moderate	5 (16.1)	2 (6.3)	
A lot	3 (9.7)	1 (3.1)	
Very much	0	0	
**Sleep disturbance**			p=0.232^a^
None	13 (41.9)	21 (65.6)	
Mild	10 (32.3)	8 (25)	
Moderate	5 (16.1)	3 (9.4)	
Severe	2 (6.5)	0	
Very severe	1 (3.2)	0	
**Would recommend to others**			
Yes	31 (100)	32 (100)	
No	0	0	
**Satisfied with intervention**			
Yes	31 (100)	32 (100)	
No	0	0	

*^a^Chi-square test.*

The results obtained with the SF-12 survey are shown in Table 6 . Similar values were recorded in both groups, with medians of 44.3 ± 12.8 for the S-ICD and 48.8 ± 9.8 for the TV-ICD on the physical health scale. The mental health scale gave medians of 45.9 ± 13.7 for the S-ICD and 50.8 ± 10.3 for the TV-ICD.

**Table 6:** – SF-12 survey results in patients implanted with their first ICD

	Subcutaneous N=32				Transvenous N=32				p value

	Median	IQR	Minimum	Maximum	Median	IQR	Minimum	Maximum	
Physical health scale	44.3	12.8	27.4	56.7	48.8	9.8	31.6	62.6	p=0.302^a^
Mental health scale	45.9	13.7	26.3	56.8	50.8	10.3	18.7	55.5	p=0.345^a^
Physical functioning scale	47.9	17.2	22.1	56.5	56.5	15.1	22.1	56.5	p=0.099^a^
Physical limitation scale	29.5	9.2	20.3	29.5	29.5	9.2	20.3	29.5	p=0.656^a^
Pain scale	57.4	0	16.7	57.4	57.4	0	37.1	57.4	p=0.150^a^
General health scale	44.7	10.8	18.9	62	55.5	10.8	18.9	62	p=0.354^a^
Vitality scale	57.8	30.2	17.6	67.9	67.9	20.2	27.6	67.9	p=0.157^a^
Emotional limitation scale	56.6	9.2	16.2	56.6	56.6	10.1	26.3	56.6	p=0.317^a^
Social function scale	22.5	0	11.3	22.5	22.5	0	11.3	22.5	p=0.263^a^
Mental health scale 2	64.5	18.3	21.9	70.6	64.5	18.3	21.9	70.6	p=0.163^a^

*IQR: Interquartile range.^a^Mann-Whitney U test.*

Tables 7 and 8 show the results of the ICD QoL questionnaire and the SF-12 survey, respectively, in patients implanted with an S-ICD after removal of a transvenous system. In terms of intraoperative pain assessments, no patients in the S-ICD group reported pain, compared with 57% who reported pain with transvenous systems. This pain was moderately severe, at most. No statistically significant differences were found in perioperative, postoperative or long-term pain. Likewise, there were no differences between ICD types as regards sleep disturbances or degree of aesthetic discomfort. All the patients were satisfied with the intervention and would recommend having the device implanted if necessary. When asked which type of ICD they would recommend, all but one of them preferred the subcutaneous system.

**Table 7:** – ICD QoL questionnaire results in patients implanted with an S-ICD following removal of a TV-ICD

	Subcutaneous N=7	Transvenous N=7	p value
**Intraoperative pain**			p=1^a^
No pain	7 (100)	3 (42.9)	
Mild	0	2 (28.6)	
Moderate	0	2 (28.6)	
Severe	0	0	
Very severe	0	0	
**Perioperative pain**			p=0.224^a^
No pain	4 (57.1)	4 (57.1)	
Mild	2 (28.6)	1 (14.3)	
Moderate	1 (14.3)	2 (28.6)	
Severe	0	0	
Very severe	0	0	
**Postoperative pain**			p=0.659^a^
No pain	6 (87.1)	6 (87.1)	
Mild	1 (14.3)	1 (14.3)	
Moderate	0	0	
Severe	0	0	
Very severe	0	0	
**Current pain**			p=0.659^a^
No pain	6 (87.1)	6 (87.1)	
Mild	1 (14.3)	1 (14.3)	
Moderate	0	0	
Severe	0	0	
Very severe	0	0	
**Aesthetic discomfort**			p=0.717^a^
None	5 (71.4)	4 (57.1)	
Mild	1 (14.3)	2 (28.6)	
Moderate	1 (14.3)	1 (14.3)	
A lot	0	0	
Very much	0	0	
**Activities of daily living limited**			p=0.427^a^
None	5 (71.4)	5 (71.4)	
A little	2 (28.6)	2 (28.6)	
Moderate	0	0	
A lot	0	0	
Very much	0	0	
**Sleep disturbance**			p=0.350^a^
None	5 (71.4)	4 (57.1)	
Mild	0	0	
Moderate	1 (14.3)	3 (42.9)	
Severe	1 (14.3)	0	
Very severe	0	0	

*^a^Chi-square test.*

**Table 8:** – SF-12 survey results in patients implanted with an S-ICD following removal of a TV-ICD

	Median	IQR	Minimum	Maximum
Physical health scale	51.3	5.3	30.5	52.9
Mental health scale	46	4.8	40.2	51.3
Physical functioning scale	56.5	0	22.1	56.5
Physical limitation scale	29.5	0	20.3	29.5
Pain scale	57.4	10.1	47.3	57.4
General health scale	55.5	10.8	29.6	62
Vitality scale	57.8	0	27.6	67.9
Emotional limitation scale	56.6	10.1	16.2	56.6
Social function scale	22.5	0	11.3	22.5
Mental health scale 2	58.4	6.1	58.4	64.5

*IQR: Interquartile range.*

## Discussion

This study demonstrates that there are no statistically significant differences in impact on quality of life in patients with an S-ICD versus those with a TV-ICD. Moreover, specific evaluation of variables that prove more controversial when assessing and choosing the type of system to implant, such as parameters related to the surgical procedure or technical specifications of the device, likewise showed no significant differences between the two patient groups.

Previous study results regarding the impact of ICD on patients’ quality of life are contradictory. Whereas some studies found that quality of life worsened or did not change significantly after ICD implantation,^27^ others noted gradual improvement.^28^ However, only one study to date has assessed quality of life in S-ICD patients. EFFORTLESS QoL^19^ is an international multicentre registry substudy that compared quality of life in S-ICD patients against a historical TV-ICD patient population. No statistically significant differences were found in quality of life, as evaluated by the SF-12 survey.

Our study results resemble those of Pedersen et al.^19^ Our results with the SF-12 quality-of-life survey, administered to patients implanted with an ICD for the first time, showed no differences in either the mental or the physical health scales.

Ours is the first study evaluating quality-of-life impact in S-ICD patients, emphasizing the analysis of potential features (involving both surgical technique and type of system implanted) that might influence the results. Many studies have now demonstrated the efficacy and safety of this type of ICD compared with conventional devices. This has allowed the indications to be expanded, and has contributed to approval by medical staff. Even today, however, some uncertainty is commonly encountered among patients and, especially, health professionals, when it comes to indicating and choosing this type of system in selected patients, mainly on account of the size difference, the different location, and the implantation technique. In an attempt to address these issues, we designed a specific questionnaire and compared our S-ICD patient population against a TV-ICD group for whom antibradycardia therapy, antitachycardia pacing, and resynchronisation therapy were not indicated (i.e. potential S-ICD candidates). Patients were matched by age, sex, and body mass index. We regard these as potential confounding variables when evaluating quality-of-life impact according to the type of system implanted.

It is apparent that some degree of pain occurred in general with both systems, with perioperative pain more severe among S-ICD patients. There were no differences in severity of intraoperative pain or long-term pain. Somewhat inconsistent postoperative management of these patients may have influenced this result, as these patients are admitted to the ward and cared for by different medical and nursing teams. Nevertheless, these findings are undoubtedly relevant, and S-ICD recipients should therefore be given stronger perioperative analgesia. No statistically significant differences were found when aesthetic discomfort, sleep disturbances, and daily activities were compared between the two groups.

Another novel aspect of this study is the assessment of perceived quality of life in patients who have had both types of therapy. These patients reported more severe intraoperative pain, aesthetic discomfort, and sleep disturbances with the transvenous system, although these differences are not statistically significant, possibly because of the group’s small sample size (7 patients). This was undeniably a biased population, because the subcutaneous system was implanted after a complication had occurred with the transvenous system. The parameters assessed, however, such as severity of pain during the surgical intervention, sleep disturbances, and aesthetic discomfort, are unrelated to the complications that arose with the conventional device; these issues are, thus, potentially independent of the negative repercussions of the system.

These data demonstrate that the different size and location of the S-ICD do not negatively influence patient quality of life.

On the other hand, our study provides the first data on patient safety and comfort/pain during surgical interventions to implant an S-ICD using a conscious sedation protocol, managed entirely by an electrophysiology team (medical and nursing staff). Although TV-ICDs are now mainly implanted under local anesthesia, S-ICDs are implanted under general anesthesia at most hospitals. In the largest multicentre study to date, 63% of sites implanted S-ICDs under general anaesthesia.^5^ This resource has limited availability at most sites. It involves organizational effort, more staff during the intervention, and higher healthcare costs. The literature contains several clinical case series describing experiences with S-ICD implantation under sedation, with strict supervision by expert anesthetists. The study by Essandoh et al.^29^ retrospectively analyzed the efficacy and safety of S-ICD implantation under anesthetist-supervised sedation, in a total of 10 selected patients. No hemodynamic or respiratory complications were reported.

The safety and efficacy of conscious sedation have already been demonstrated in patients undergoing ablation for atrial fibrillation,^20^ and this method is routinely used in our laboratory. For S-ICD implantation, we used a sedation protocol adapted for this type of procedure, in order to ensure adequate analgesia for the patients throughout the entire intervention. No complications were recorded during the procedure. It should be noted that 100% of patients implanted with both types of system described a complete absence of pain during S-ICD implantation, whereas fewer than half of those patients reported not having felt any pain during the TV-ICD procedure.

### Limitations

One limitation of the study is potential interviewer bias. In order to prevent this, surveys were administered over the telephone by the same blinded investigator. To avoid recall bias in the interview subject, only patients implanted with an ICD in the last 2 years were included.

The control population consisted of TV-ICD patients matched by age, sex, and body mass index. These are variables that we think might influence patients’ response regarding degree of discomfort/satisfaction with the S-ICD versus the TV-ICD. Nevertheless, other variables not controlled for by the study design, such as ICD indication, type of heart disease, or functional class, as well as pre-implantation quality of life, could have influenced these patients’ quality of life, and thus affected assessment of the specific impact of the ICD. However, the absence of statistically significant differences in baseline patient characteristics lessens this potential limitation considerably.

A possible limitation of this study is the lower prevalence of discharges suffered by the S-ICD group (5.1% versus 17.9%), which could have some influence on the perception of quality of life when analyzing this subgroup of patients. However, the prevalence of discharges in both groups was low (11%). We thus believe that this has not significantly influenced the overall results of our study.

Lastly, the sample was small in size, being obtained from just one hospital, thereby limiting the statistical power needed to detect differences. However, our quality-of-life data resemble those published recently from a larger population.^5^

## Conclusions

The type of ICD implanted does not significantly influence patients’ perception of mental or physical quality of life. Our study demonstrates that differences in the surgical procedure (both location and surgical technique) or type of system implanted (such as weight and size) do not have a negative impact on patient quality of life. On the other hand, these findings suggest that the S-ICD can be safely implanted under conscious sedation by an electrophysiology team. Larger, randomized studies are needed to compare against and confirm these results.

### Key Points

#### What is already known about this subject?

- The subcutaneous ICD has been shown to be similar in efficacy to the conventional ICD at preventing sudden cardiac death.
- The subcutaneous ICD is an alternative to the transvenous ICD in patients not requiring antibradycardia, antitachycardia, or cardiac resynchronisation pacing; patients with difficult venous access; young patients; or following removal of a conventional ICD because of infection.
- The subcutaneous ICD employs a different surgical technique from the conventional ICD, and the generator is larger and heavier than in current transvenous systems.

#### What does this study add?

- There are no significant differences in mental or physical quality of life among a Spanish population of patients with subcutaneous or transvenous ICDs.
- Differences in surgical technique or type of system implanted do not negatively affect patient quality of life.
- Patients implanted with a subcutaneous ICD after having a transvenous ICD removed because of complications assess the new device positively.
- The subcutaneous ICD can safely be implanted under conscious sedation by an electrophysiology team.

## * Supplemental Materials

For additional information, please click here.
